# Molecular Phylogeny of Asian *Meconopsis* Based on Nuclear Ribosomal and Chloroplast DNA Sequence Data

**DOI:** 10.1371/journal.pone.0104823

**Published:** 2014-08-12

**Authors:** Yu-Cheng Liu, Ya-Nan Liu, Fu-Sheng Yang, Xiao-Quan Wang

**Affiliations:** 1 State Key Laboratory of Systematic and Evolutionary Botany, Institute of Botany, Chinese Academy of Sciences, Beijing, China; 2 University of Chinese Academy of Sciences, Beijing, China; The National Orchid Conservation Center of China; The Orchid Conservation & Research Center of Shenzhen, China

## Abstract

The taxonomy and phylogeny of Asian *Meconopsis* (Himalayan blue poppy) remain largely unresolved. We used the internal transcribed spacer (ITS) region of nuclear ribosomal DNA (nrDNA) and the chloroplast DNA (cpDNA) *trnL-F* region for phylogenetic reconstruction of *Meconopsis* and its close relatives *Papaver*, *Roemeria*, and *Stylomecon*. We identified five main clades, which were well-supported in the gene trees reconstructed with the nrDNA ITS and cpDNA *trnL-F* sequences. We found that 41 species of Asian *Meconopsis* did not constitute a monophyletic clade, but formed two solid clades (I and V) separated in the phylogenetic tree by three clades (II, III and IV) of *Papaver* and its allies. Clade V includes only four Asian *Meconopsis* species, with the remaining 90 percent of Asian species included in clade I. In this core Asian *Meconopsis* clade, five subclades (Ia–Ie) were recognized in the nrDNA ITS tree. Three species (*Meconopsis discigera*, *M. pinnatifolia*, and *M. torquata*) of subgenus *Discogyne* were imbedded in subclade Ia, indicating that the present definition of subgenera in *Meconopsis* should be rejected. These subclades are inconsistent with any series or sections of the present classifications, suggesting that classifications of the genus should be completely revised. Finally, proposals for further revision of the genus *Meconopsis* were put forward based on molecular, morphological, and biogeographical evidences.

## Introduction

The genus *Meconopsis* Vig. includes about 50–60 species that are distributed mainly in the Qinghai-Tibetan plateau (QTP) [1]–[4] and is a symbol of the Himalayan alpine flowers. The only European species, *Meconopsis cambrica* is distributed in the humid and shady deciduous forests of Ireland and from south-west England to Northern Spain. The genus is well known as the ‘Himalayan blue poppy’ and has fascinated the Western world because of its attractive flowers [1]–[5]. Species of this genus can also be found in some European gardens, since they were introduced in these regions about two centuries ago. However, the taxonomy and phylogeny of the genus of this famous garden plant species remain largely unresolved [2], [3], [6].

The genus *Meconopsis* was founded in 1814 on the basis of the single European species *Papaver cambrica* L. [1]. This species is different from typical *Papaver* species because of the presence of a short style and the complete absence of a sessile stigmatic disc surmounting the ovary. With the description of more species of *Meconopsis*, the generic limits between *Meconopsis* and its close relatives have become more unclear. For example, nine species of *Meconopsis* have been incorrectly included in the closely related genera such as *Papaver*, *Cathcartia*, and *Stylophorum* [1], [7], [8]. The monotypic *Stylomecon* was described originally as *Meconopsis heterophylla* on the basis of its obvious style; subsequently, Kadereit and Baldwin [7] suggested that it should be included in *Papaver*. The taxonomic status of *Meconopsis villosa* (*Cathcartia villosa*) is also yet unknown [7], [9]. In fact, parallel evolution of the style in the subfamily Papaveroideae was detected by conducting phylogenetic and ontogenetic analyses on the plant group [10]–[11].

Since the first detailed taxonomic study of *Meconopsis* [12], several influential but controversial classification systems for the genus have been proposed [1]–[2], [13]–[15]. Much of the differences between systems can be attributed to the definition of primary classification of the genus. For example, in the system of Prain [13], two sections were recognized on the base of the pubescence characters. This treatment was substantially accepted by Fedde [16], but the sections were treated as subgenera and the minor groups were raised to the rank of sections. The monotypic section *Cambricae* included the only European *Meconopsis* (*M. cambrica*). Subsequent authors emphasized the characters of style. For example, in the system of Taylor [1], the genus *Meconopsis* was treated as two subgenera (*Eumeconopsis* and *Discogyne*) on the based of the shape of the style. In the subgenus *Eumeconopsis*, three sections were determined primarily by habit, flower-colour and pubescence characters, and the monotypic section *Cambricae* of Fedde [16] was retained. Also, the monotypic genus *Cathcartia*, firstly described in 1851 based on the unique capsule-valves extending beyond the base of the style, was referred to *Meconopsis* [1]. Except for the definition of subgenera, Taylor’s classification system was substantially re-organized by Wu and Chuang in 1980 [2]. In this system, five sections and nine series were recognized based on characters of inflorescence, stem, leaf and root, with section *Cambricae* including *M. cambrica* and nine species of Asian *Meconopsis*.

Molecular phylogenetic analysis showed that *Meconopsis*, *Papaver*, *Roemeria*, and *Stylomecon* were included in ‘Old World Papaveroideae’ (OWP) in the subfamily Papaveroideae [17], and neither *Meconopsis* nor *Papaver* formed a monophyletic clade. Further, the phylogenetic relationships of the main clades in the OWP were unresloved. A subsequent phylogenetic analysis (an RFLP analysis of chloroplast DNA (cpDNA) *trn*K) of the OWP indicated that 14 species of Asian *Meconopsis* formed two clades, but one clade was weakly supported (bootstrap support value, <50). *Meconopsis cambrica*, the only European species of the genus, was placed in the third clade that included all sampled species of *Papaver*, *Stylomecon*, and *Roemeria* [18], with a bootstrap support lower than 50%. Interestingly, if the European *Meconopsis* was excluded from the phylogenetic analysis, the topology of the main clades in the OWP would have changed remarkably [18]. When the basalmost Asian *Meconopsis* clade was not included in the phylogenetic analysis [10], the topology of the main clades in the OWP was also considerably different from that reported previously [17]–[18]. Although the phylogenetic position of the western European endemic *M. cambrica* was determined recently [19], the relationships of the main clades of OWP remain unresolved. Also, the Asian *Meconopsis* accessions in the analyses were obtained from Genbank, for which getting information concerned is difficult; therefore, the phylogenetic relationships in the clades of Asian *Meconopsis* were seldom discussed [19]. The above-mentioned phylogenetic analyses suggest the importance of field sampling and sampling strategy in phylogenetic analysis of *Meconopsis*. Many species of *Meconopsis* are rare and endangered species that are distributed in the geographically challenged area (QTP); therefore, collecting samples from the wild is a challenging task. Nevertheless, sufficient sampling should be necessary to reconstruct a reliable phylogeny of *Meconopsis*, considering the complicated relationships between the genus and its close relatives.

In this study, 42 species representing all series of *Meconopsis* [1], [2] and representatives of all sections in *Papaver* [19]–[26] were sampled to reconstruct a phylogeny of the OWP. One species each of the oligotypic genus *Roemeria* and the monotypic genus *Stylomecon* were also included in the analysis. This study aimed to (1) elucidate the phylogenetic relationships between *Meconopsis* and its close relatives and (2) provide a proposal for the taxonomic treatment for Asian *Meconopsis*.

## Materials and Methods

### Plant materials

Our sample included 42 species of *Meconopsis* (of the approximately 50–60 species) representing all sections and series in the genus [1], [2] and 30 species of *Papaver* (of the approximately 80 species) representing all the 11 sections of the genus (Table S1) [19]–[26]. Of the 112 individuals from the 42 species of *Meconopsis*, 88 were newly sequenced using internal transcribed spacer (ITS) of nuclear ribosomal DNA (nrDNA) and cpDNA *trnL-F* regions, and the sequences of the remaining 24 individuals were obtained from Genbank (Table S1). Over half of the *Meconopsis* species were sampled by including more than one individual from one or several populations. Sequences of *Papaver* were mainly obtained from Genbank. Previous studies [10], [17]–[18] indicated that insufficient sampling can influence the topologies of the OWP phylogenetic trees; therefore, 19 species representing 12 genera of Papaveraceae *s.s.* were included in the phylogenetic analysis. *Fumaria densiflora* and *Discocapnos mundtii* from family Fumariaceae *s.s.* were sampled as outgroups, on the basis of recent studies on the phylogeny of family Papaveraceae *s.l.*

### DNA extraction, PCR amplification, and sequencing

Total genomic DNA was isolated from silica gel-dried leaves by using a modified cetyltrimethylammonium bromide (CTAB) method [27]. Amplification of the nrDNA ITS and the *trnL-F* regions followed Yang *et al.* [28]. The PCR products were purified using Gel Band Purification Kit (Tiangen Biotech, Beijing, China), and then sequenced using the ABI Prism BigDye Terminator Cycle Sequencing Ready Reaction Kit (Applied Biosystems, Foster City, CA, USA) on an ABI PRISM 3730xl analyzer (Applied Biosystems). Direct sequencing of ITS region produced double peaks in chromatograms of six individuals. Molecular cloning and sequence analyses of the ITS region detected sequence polymorphisms in these individuals. Further phylogenetic analyses combined with morphological evidence suggest that all intra-individual polymorphisms can be attributed to inter-species hybridization other than to ITS paralogs. Phylogenetic analyses of ITS region in the present study mainly aim to resolve general relationships in the genus; therefore, the hybrids were excluded from the phylogenetic analyses.

### Phylogenetic analysis

The DNA sequences were aligned using the default parameters in Clustal X [29], and then refined manually in BioEdit. To reconstruct the phylogeny of *Meconopsis* and its close relatives, Maximum parsimony (MP) and Bayesian inference (BI) analyses for the nrDNA ITS and cpDNA *trnL-F* regions were implemented in software packages PAUP* 4.0b10 [30] and MrBayes version 3.1.2 [31], respectively. For the MP analysis, all characters were weighted equally and treated as unordered, with gaps considered as missing. Heuristic searches were conducted using 1,000 replicates of random addition, tree-bisection-reconnection branch swapping, the MULTREES option, and a maximum of 1,000 trees saved per round. The confidence of clades in the MP trees was estimated by performing bootstrap analysis with 1,000 replicates by using the heuristic search. Before performing the BI analysis, the best evolutionary models of the two regions were determined using the Akaike Information Criteria implemented in MrModeltest 2.3 [32]. The GTR+I+G model was chosen for the nrDNA ITS and *trnL-F* regions. Two separate runs of four Markov chains for 10,000,000 generations were applied for each data set, sampling one tree per 1000 generations. The 50% majority-rule consensus tree was constructed after removing the first 2,000 trees. MCMC convergence was explored by examining the Potential Scale Reduction Factor (PSRF) convergence diagnostics for all parameters in the model. Posterior probabilities were calculated for sampled trees.

## Results

### Sequence characterization and phylogenetic analyses

**Figure 1:**
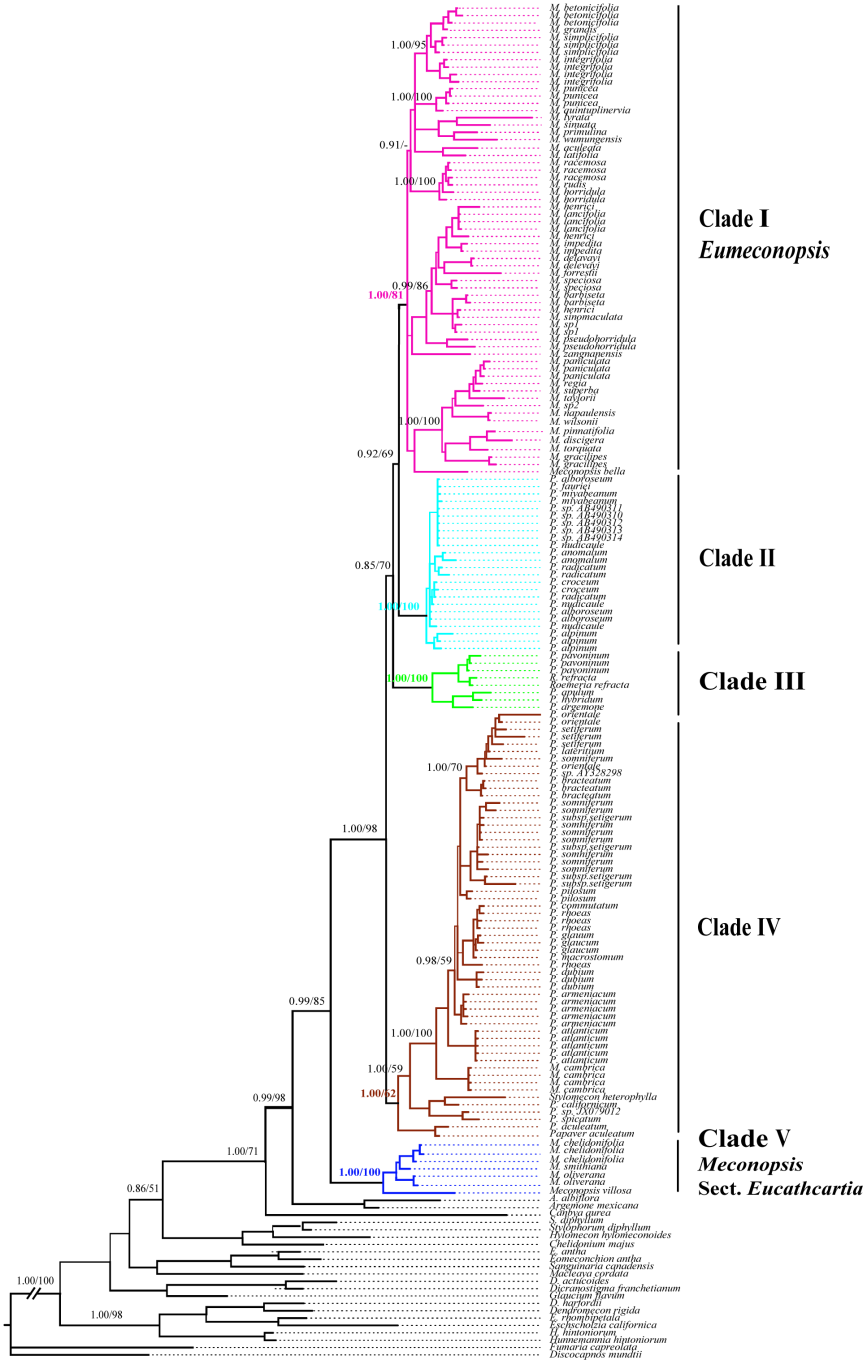
The Bayesian tree of Meconopsis constructed using the internal transcribed spacer region of nuclear ribosomal DNA (nrDNA ITS). Numbers on the branches denote the Bayesian posterior probabilities and the bootstrap values for maximum parsimony (MP) for the main clades.

**Figure 2:**
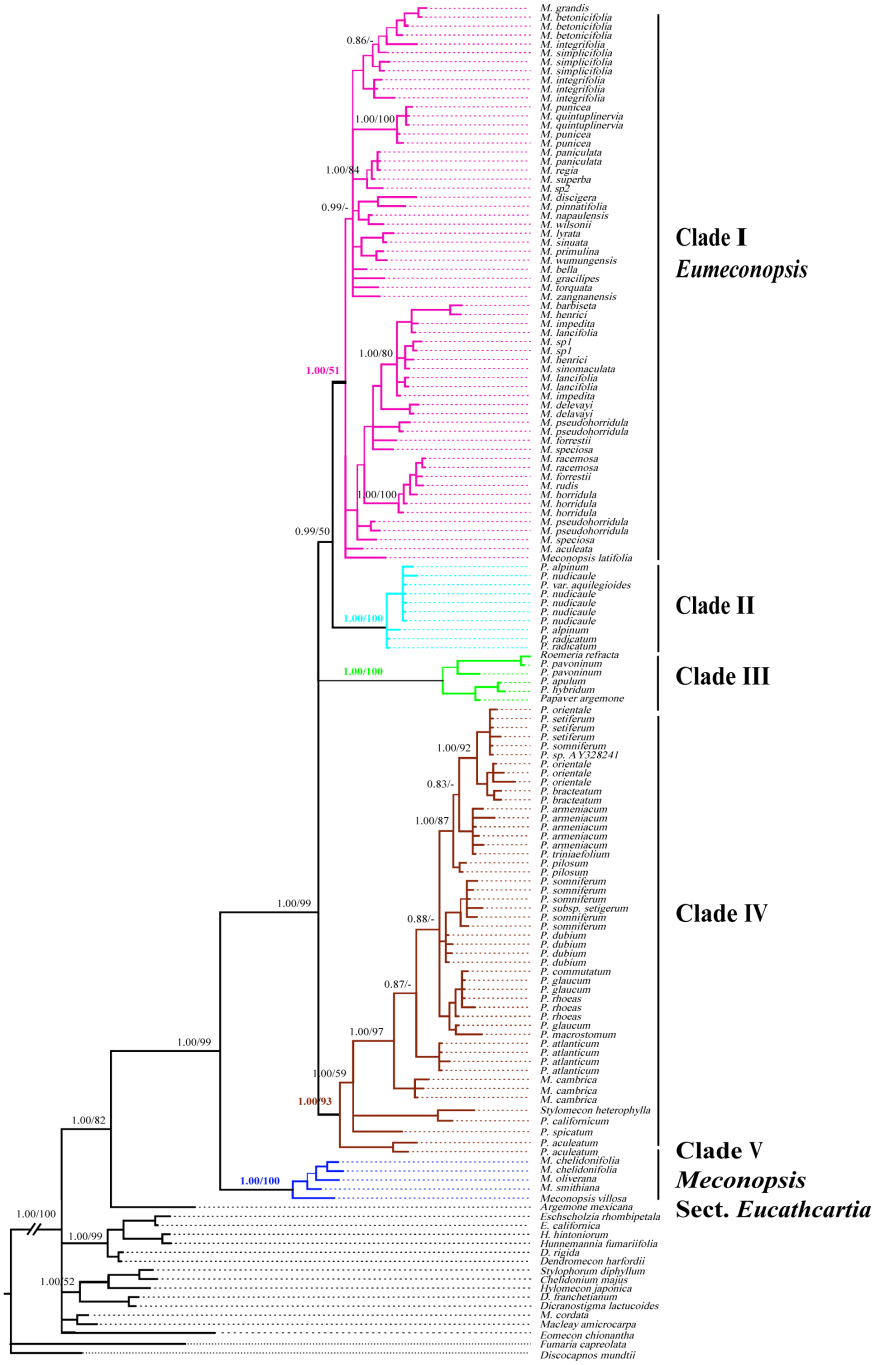
The Bayesian tree of *Meconopsis* inferred from the trn*L-F* fragment. Numbers on the branches denote the Bayesian posterior probabilities and the bootstrap values for maximum parsimony (MP) for the main clades.

The aligned nrDNA ITS sequence data matrix was 866 bp in length, with 360 potentially parsimony-informative and 87 uninformative variable characters. The aligned cpDNA *trnL-F* matrix consisted of 964 characters, of which 280 were variable and 194 were potentially parsimony informative. The BI consensus trees of the nrDNA ITS and cpDNA *trnL-F* matrices are shown in Figures 1 and 2, respectively. The topologies of most parsimony trees (not shown) reconstructed with the two matrices were nearly identical to those shown in Figures 1 and 2 except for some terminal branches. The monophyly of the Old World clade (including *Meconopsis*, *Papaver*, *Stylomecon*, and *Roemeria*) in subfamily Papaveroideae was strongly supported in Figure 1 (Bootstrap support (BS) = 85; Bayesian posterior probability (PP) = 0.99) and Figure 2 (BS = 99; PP = 1.00), with New World clade (*Argemone*) as a sister group. Five main clades (I–V) recognized in the nrDNA ITS gene tree were well supported by the cpDNA *trnL-F* gene tree, and neither *Meconopsis* nor *Papaver* was supported as monophyly. The monotypic genera *Stylomecon* and oligotypic genera *Roemeria* were nested in clades of *Papaver* or *Meconopsis*.

**Figure 3:**
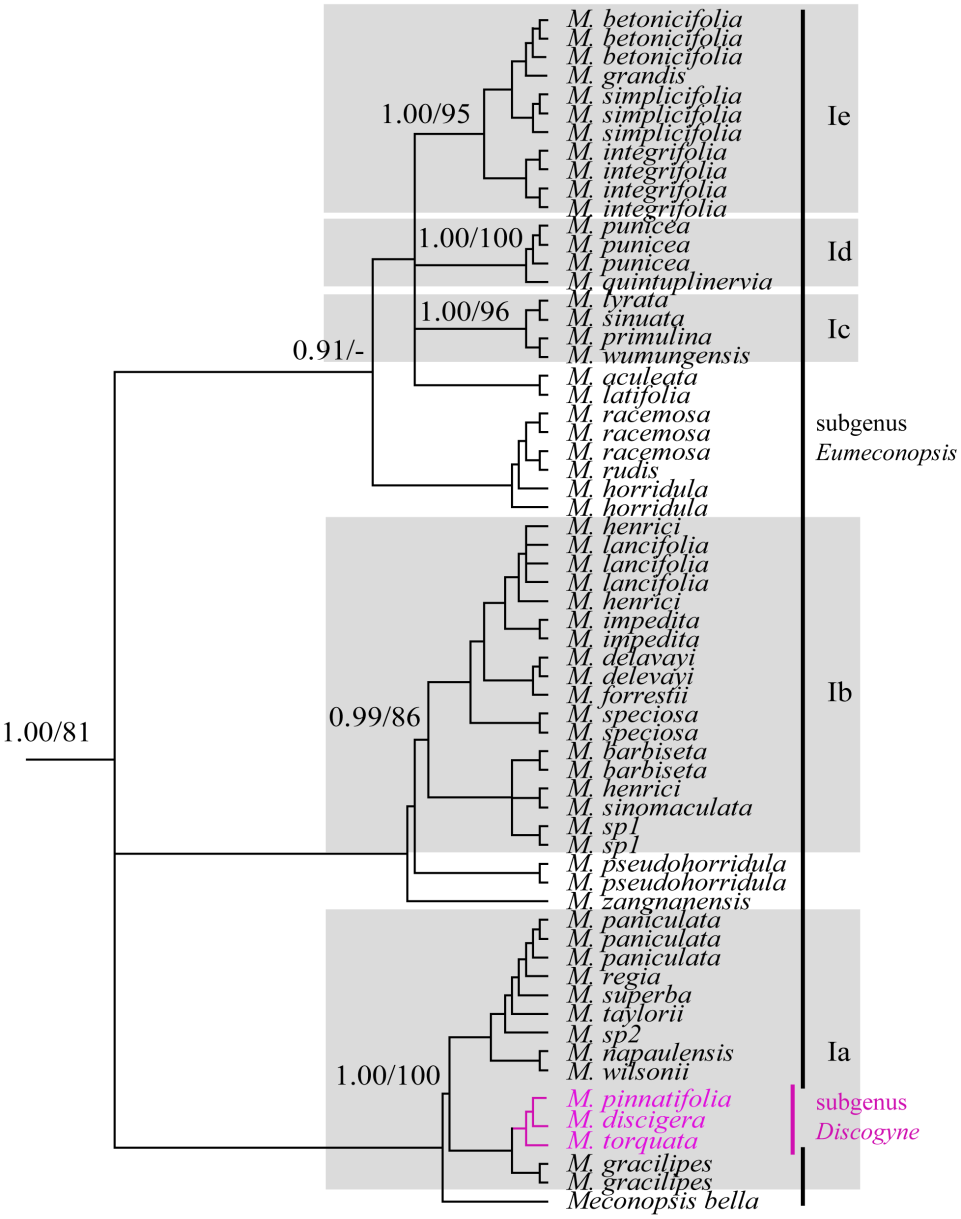
The Bayesian tree of clade I from **Figure 1**. Numbers on the branches denote the Bayesian posterior probabilities and the bootstrap values for maximum parsimony (MP) and the Bayesian posterior probabilities for the main clades.

Clade I comprised 37 species of *Meconopsis*, and only five species (*M. cambrica* and four species of clade V) of the genus were placed outside. Plants of this clade are only found in the areas from the Himalayas to the Hengduan Mountains. Clade I of the ITS tree revealed five well supported subclades (Ia–Ie; Figure 3), which were not well resolved in the *trnL-F* gene tree (Figure 2). Seven species of the subsection *Eupolychaetia* [1] and three species of the subgenus *Discogyne* formed subclade Ia (Figures 1 and 3), and the remaining four subclades were not consistent with any sections or series of the present classifications [1], [2] (Table S2).

All sampled species of *Papaver* sect. *Meconella* formed a well-supported clade II, and species of this section were distributed in the arctic and subarctic mountains (outside the QTP). Clade III consisted of *Roemeria refracta* and all the four species in *Papaver* section *Argemonidium*. Clade IV comprised *Meconopsis cambrica*, *Stylomecon heterophylla*, and 16 species of *Papaver*. The 16 species of *Papaver* represented nine of the 11 sections except sections *Meconella* and *Argemonidium*. *Papaver aculeatum*, the only poppy native to Eastern South Africa, and two species (*Stylomecon heterophylla* and *Papaver californicum*) endemic to California occupied the basal positions in this clade. *Meconopsis cambrica* from Western Europe was included in a separate branch. The remaining 13 species of *Papaver* were distributed in North Africa and Europe to South-West and Central Asia, with species diversity centre located in the Mediterranean area where 32 species of *Papaver* were distributed [33]. Clade V consisted of four *Meconopsis* species that were sporadically distributed in the narrow area from south Himalayas to east Hengduan Mountains, extending to the mountains of Central Asia.

## Discussion

### Phylogeny of Asian *Meconopsis*

Previous studies have indicated that *Meconopsis*, *Papaver*, *Roemeria*, and *Stylomecon* formed a well-supported clade, the Old World clade of the subfamily Papaveroideae. This group was divided into four parallel clades, but only a small clade, including species of *Papaver* section *Argemonidium* and *Roemeria*, was well supported (Figure 1 in [11]). In this study, the phylogenetic analyses of the nrDNA and cpDNA sequences combined with extensive sampling showed that neither *Meconopsis* nor *Papaver* is monophyletic (Figures 1 and 2), supporting the results of Kadereit *et al.* [18] and Carolan *et al.* [10]. Most importantly, our results identified five well-supported OWP clades, which were well resolved in the gene trees reconstructed with the nrDNA ITS and cpDNA *trnL-F* sequences (Figures 1 and 2). The two Asian *Meconopsis* clades (I and V) were separated in the phylogenetic trees by three clades of *Papaver* and its allies, with clades I and V occupying the shallow-most and basal-most positions in the OWP. The only European *Meconopsis* species (*M. cambrica*) were nested in clade IV comprising 16 species of *Papaver*. In addition, in clade I, which contained about 90% species of Asian *Meconopsis*, five well-supported subclades were recognized in the nrDNA ITS tree. The three clades of *Meconopsis* are discussed below according to morphology and biogeography.

Clade I, *Meconopsis* section *Eumeconopsis*. This core *Meconopsis* clade comprised 37 species of Asian *Meconopsis*, including 34 species of subgenus *Eumeconopsis* and three species of subgenus *Discogyne* (Figures 1–3). Only five species of subgenus *Eumeconopsis* were placed outside this clade: *Meconopsis cambrica* in clade IV and the four species in clade V. Species of *Meconopsis* in these two clades are characterised by four-petalled yellow flowers with uniform colour of petals and stamens, whereas the two organs in species of clade I often display different colour. Further, species of *Meconopsis* in clade I can be differentiated from typical *Papaver* on the basis of the presence of a typical style and lack of a stigmatic disc.

**Figure 4:**
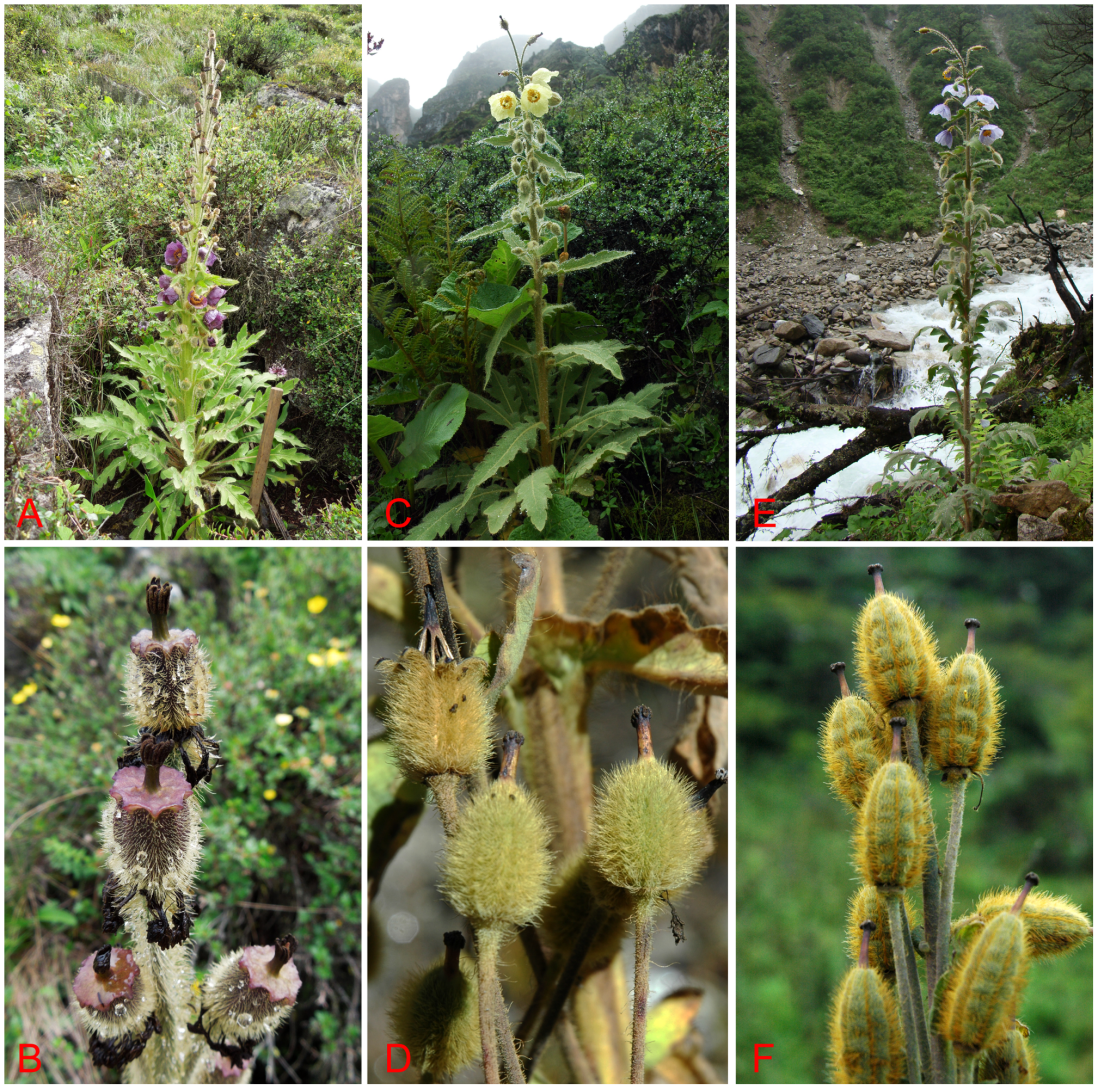
The illustrations show representative species from subclade Ia. (A, B) Plant and fruit of *Meconopsis pinnatifolia* (subgenus *Discogyne*); (C, D) *M. paniculata* (subgenus *Eumeconopsis*); (E, F) *M. wilsonii* (subgenus *Eumeconopsis*).

Our phylogenetic results indicated that three species (*M. discigera*, *M. pinnatifolia*, and *M. torquata*) of subgenus *Discogyne* were imbedded in clade I that included 34 species of subgenus *Eumeconopsis* (Figures 1–3), which are inconsistent with recent classifications [1], [2] in which *Meconopsis* was divided into two subgenera on the basis of ovary characters. The subgenus *Discogyne* was first recognised by Taylor [1] on the basis of the distinct stylar disc surmounting the ovary (Figure 4). It includes four species distributed in the eastern Himalayas [34], and two new species (*M. manasluensis* and *M. bhutanica*) described recently should be included in this subgenus because of the presence of a stylar disc [3], [35]. Considering the unique structure, Taylor thought that this group could be treated as a separate genus; however, the habit was similar to some more typical *Meconopsis* species found in the same distribution area.

**Figure 5:**
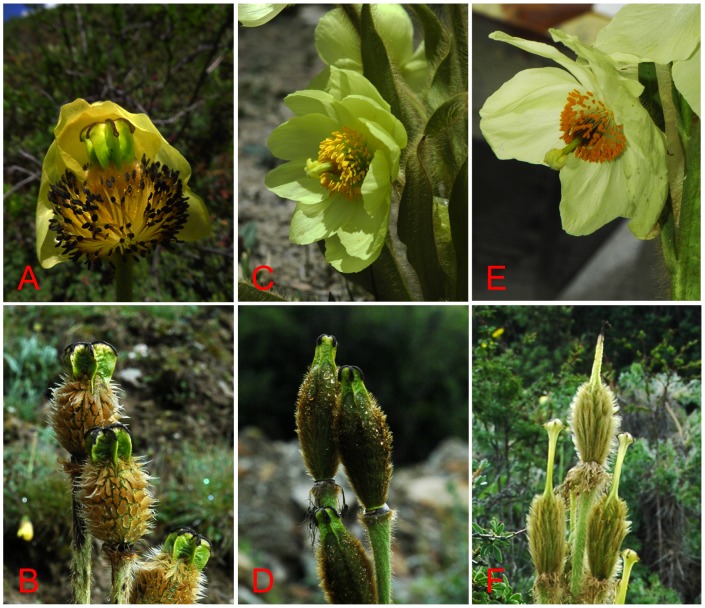
The illustrations show morphological diversity of styles within *Meconopsis integrifolia*. A and B, C and D, and E and F represent plants from the same population, respectively.

In fact, except for the distinct stylar disc surmounting the ovary, species of subgenus *Discogyne* are morphologically quite similar to the others in subclade Ia (Figure 4). Both are monocarpic herbs up to 1.0–2.0 m in height, with indumentum sparsely to densely bristly throughout whole plant. Leaves are basal and cauline, lamina lanceolate or elliptic-oblong, pinnatilobate to pinnatisect. Inflorescence is racemose or paniculate (Figure 4). Recently, Kadereit and Erbar [11] confirmed that the obvious stylar disc initially used as a diagnostic trait to distinguish genera in the OWP or subgenera in *Meconopsis* has evolved several times independently. Also, our field investigations found that style length and the presence or absence of stylar disc varied remarkably at population levels in some species of *Meconopsis*, such as *M. integrifolia* (Figure 5). Thus, it is not appropriate to delimitate two subgenera of the genus *Meconopsis* on the basis of stylar disc, which has experienced parallel evolution in the lineages of the OWP.

**Figure 6:**
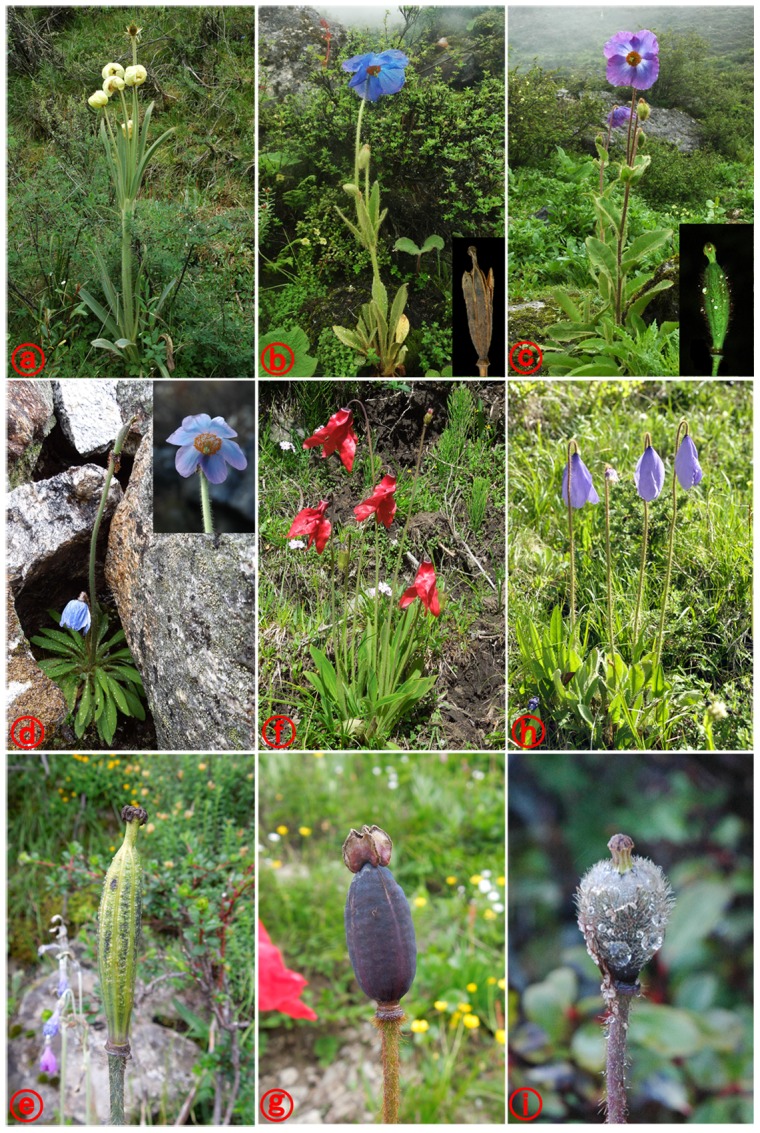
The illustrations show plants of subclade Ie (a–e) and Id (f–i). (a) *Meconopsis integrifolia*; (b) *M. grandis*; (c) *M. betonicifolia*; (d, e) *M. simplicifolia*; (f, g) *M. punicea*; (h, i) *M. quintuplinervia*.

Although the present classifications [1], [2] of *Meconopsis* were not recognized by our molecular results, there are some consistencies between subclades of clade I and sections/series of Taylor [1] (Table S2). For instance, subsection *Eupolychaetia* is consistent with subclade Ia except for species of subgenus *Discogyne*. Subclade Ic includes *M. lyrata*, *M. primulana*, *M. wumungensis*, and *M. sinuate*, which is substantially congruent with series *Primulinae*. The only exception is *M. sinuate*, which was placed in series *Aculeatae* by Talyor [1], should be transferred to series *Primulinae* on the base of our molecular evidence (Figures 1–3). Series *Simplicifoliae* [1], [2] (Table S2) includes *M. punicea*, *M. quintuplinervia*, and *M. simplicifolia*. But our results suggest that the last species should be transferred to series *Grandes*. In fact, the semi-drooping flower and narrowly ellipsoid-oblong capsule (Figure 6d, 6e) of *M. simplicifolia* are quite similar to those of series *Grandes* (*M. betonicifolia* and *M. grandis*) (Figure 6b, 6c) rather than those of series *Simplicifoliae* (*M. punicea* and *M. quintuplinernia*) (Figure 6f–6i), which have drooping flower and ellipsoid or obovoid capsule.

Nearly all species in clade I are exclusively distributed in the unique habitats of the QTP, the roof of the world. The only exception is *Meconopsis quintuplinervia*, which is distributed eastwardly from the QTP to the adjacent Qinling Mountains. The distributions of these subclades showed clear geographic patterns. Subclade Ia included 10 species that mainly occurred in the rainy regions of the southern QTP (Himalayas), with only one species (*Meconopsis wilsonii*) extending eastwardly in the eastern QTP (Hengduan Mountains). Plants of this clade are often covered with villous or small much-branched hairs, with stem heights of 1.0 to 2.5 m. In contract, species of subclade Ib are mainly distributed in the eastern QTP, and the bristly plants are often shorter than 0.6 m. The shorter stem, thicker leaves, and covered bristles indicate the adaptation of plants to drier habitats. The small clade Ic mainly occurred in the Himalayas, and the remaining two subclades occurred in the eastern QTP. The correction between species of subclade Ie and the regional climate in the QTP has been detected by our previous phylogeographic analysis [28]; in this study, the lineage-specific distribution patterns suggest that climatic divergence driven by the rapid uplift of the QTP has led to lineage divergence in the genus *Meconopsis*.

Clade IV, Meconopsis cambrica plus *Papaver*. This clade consisted of *M. cambrica, Stylomecon heterophylla*, and representatives of nine sections in *Papaver* (i.e. sects. *Carinatae*, *Meconidium*, *Pseudopilosa*, *Rhoeadium*, *Oxytona* (*Macrantha*), *Papaver*, *Pilosa*, *Californicum*, and *Horrida*). The only European species of *Meconopsis*, *M. cambrica*, was placed within this core *Papaver* clade, which is congruent with previous molecular analysis [10]. In fact, Ernst in 1962 [36] had found that the gynaecium of *M. cambrica* has pseudodoral veins, a distinct character absent from all other species of *Meconopsis* examined but present in all sections of *Papaver* s.s. Recently, Kadereit and Erbar [11] found that gynaecium ontogeny in *Papaver* s.s is characterised by a unique “garland-like” stage, and remnants of this stage were observed in *M. cambrica*. These morphological and anatomical characters provide strong supports for a close relationship of *M. cambrica* and *Papaver* s.s [8]. The monotypic genus *Stylomecon* characterised by a distinct style considerably similar to that of *Meconopsis* was also placed within clade IV. *Stylomecon heterophylla* was originally described as *Meconopsis heterophylla*, but our results supported the findings of Kadereit and Baldwin [19] that it should be treated as *Papaver*.

Clade V, *Meconopsis* section *Eucathcartia*. This clade included *M. chelidonifolia, M. oliverana, M. smithiana,* and *M. villosa* and was strongly supported in the nrDNA ITS and cpDNA *trnL-F* gene trees (Figures 1 and 2). A clade that comprised *M. chelidonifolia* and *M. villosa* was recognized by previous molecular phylogenetic analyses; however, its relationship with other clades was poorly supported [17]. In this study, besides the two species mentioned above, *M. oliverana* and *M. smithiana* were also included in this well-supported clade, which occupies the basal position of the OWP. Morphologically, the four species are characterised by a polycarpic habit and four-petalled yellow flowers with uniform colouration of the petals and stamens; because of these features, species of clade V can be easily distinguished from other Asian *Meconopsis* species (clade I). Species of clade V are also different from typical *Papaver* species by the presence of a short style and complete absence of a sessile stigmatic disc surmounting the ovary. Because of the ‘unique’ capsule valves extending beyond the base of the style, *M. villosa* was first described as belonging to a separate genus *Cathcartia* by Hooker. This treatment was not recognised by Taylor [1], in his monography *Cathcartia villosa* and three species of *Meconopsis* (*M. chelidonifolia*, *M. oliverana*, and *M. simthiana*) were included in section *Eucathcartia*, one of the three sections in *Meconopsis* subgenus *Eumeconopsis*. Our results confirmed that these four species formed a well supported monophyletic clade.

Four species of clade V occupy the easternmost and southernmost portions of the distributional range of the genus, and these species grew in thickets at altitudes ranging from 1,500 to 2,500 m, a habit considerably different from that of the Himalayan species of Asian *Meconopsis*.

### Taxonomic implications for Asian *Meconopsis*

The present study confirmed that the Asian *Meconopsis* forms two well-supported clades, separated by two clades of *Papaver* and one clade of *Papaver* plus *Roemeria* (Figures 1 and 2). Considering the botanical and economical consequences, the generic name for the Asian *Meconopsis* species should be retained [8], and Clade V should be advanced to generic level and be named as *Cathcartia*. The well recognized five subclades in clade I suggest that series *Primulinae*, *Grandes*, and *Simplicifoliae* (Table S2) of Taylor [1] could be retained after minor revision. Series *Superbae* and *Robustae* should be united, with subgenus *Discogyne* included in this group. Series *Delavayanae* should be merged into series *Aculeatae*, in which the relationships need further investigation. Our results suggest that special attention should be paid on the texture of leaves, types of bristle covered and geographic distributions of species for future revision of the genus.

## Supporting Information

Table S1 **Sources of materials.**
(DOC)

Table S2 **Classifications of** ***Meconopsis*** **by Taylor** [1] **and Wu & Chuang** [2] **.**
(DOC)
