# Supplementary material for: Molecular Phylogeny of Asian Meconopsis Based on Nuclear Ribosomal and Chloroplast DNA Sequence Data
Source: PLoS One. 2014 Aug 12;9(8):e104823. doi: 10.1371/journal.pone.0104823 (PMC4130606; doi:10.1371/journal.pone.0104823)
Supplement: Table S1 — Sources of materials. (DOC) [file pone.0104823.s001.doc]

**Table S1. Sources of materials.**

| Taxon | ITS | *trnL-F* | Source | N |
| --- | --- | --- | --- | --- |
| *Meconopsis aculeata* | AY328263 | AY328227 | Yuan, 2002 | 1 |
| *M. bella* | AY328279 | AY328218 | Yuan, 2002 | 1 |
| *M. betonicifolia* | JQ798369  JQ798370  JQ798371 | JQ798377  JQ798378  JQ798379 | Tibet, China  Yunnan, China | 7 |
| *M. barbiseta* | KM044429  KM044430 | KM044473  KM044474 | Qinghai, China | 2 |
| *M. cambrica* | JF774139  JF774141  JF774157  JF774159 | AY328243  DQ251128  DQ251129 | Yuan, 2002  Carolan *et al.*, 2006  Valtuena *et al.*, 2011 | 4 |
| *M. chelidonifolia* | JQ798372  JQ798373  AY328300 | JQ798380  JQ798381 | Sichuan, China  Yuan, 2002 | 4 |
| *M. delavayi* | KM044431  KM044432 | KF513542  KM044475 | Yunnan, China | 4 |
| *M. discigera* | AY328277 | AY328221 | Yuan, 2002 | 1 |
| *M. forrestii* | AY328287 | AY328219 | Yuan, 2002 | 1 |
| *M. gracilipes* | KM044433  KM044434 | KF513544 | Nepal | 3 |
| *M. grandis* | KM044435 | AY328237 | Tibet, China | 2 |
| *M. henrici* | KM044437 | KF513545 | Sichuan, China | 2 |
| *M. horridula* | KM044438  KM044439 | KF513546KM044477  KM044478 | Tibet, China  Qinghai, China | 5 |
| *M. impedita* | KM044440  AY328283 | KM044479  AY328210 | Sichuan, China  Yuan, 2002 | 3 |
| *M. integrifolia* | JQ798303  JQ798312  JQ798313  JQ798299 | JQ798329  JQ798331  JQ798333  JQ798341 | Sichuan, China  Yunnan, China  Tibet, China | 11 |
| *M. lancifolia* | KM044441  KM044442  KM044443 | KF513548  KM044480  KM044481 | Yunnan, China  Sichuan, China | 4 |
| *M. latifolia* | AY328264 | AY328226 | Yuan, 2002 | 1 |
| *M. lyrata* | AY328267 | AY328215 | Yuan, 2002 | 1 |
| *M. napaulensis* | AY328269 | AY328228 | Yuan, 2002 | 1 |
| *M. oliverana* | KM044444  KM044445 | KM044482 | Shaanxi, China | 2 |
| *M. paniculata* | KM044449  KM044450  KM044451 | KM044485  KF513549 | Tibet, China | 4 |
| *M. pinnatifolia* | KM044452 | KM044486 | Tibet, China | 2 |
| *M. primulina* | AY328266 | AY328217 | Yuan, 2002 | 1 |
| *M. pseudohorridula* | KM044454  KM044455 | KF513550  KM044487  KM044488  KM044489 | Tibet, China | 4 |
| *M. punicea* | KM044446  KM044447  KM044448 | KF513551KM044483  KM044484 | Sichuan, China | 6 |
| *M. quintuplinervia* | KM044456 | KF513552KM044490 | Qinghai, China | 5 |
| *M. racemosa* | KM044457  KM044458  JX078980 | KM044491  KF513553 | Sichuan, China  Yunnan, China  Yuan, 2002 | 4 |
| *M. regia* | AY328273 | AY328224 | Yuan, 2002 | 1 |
| *M. rudis* | KM044459 | KM044492 | Yunnan, China | 1 |
| *M. simplicifolia* | JQ798382  JQ798383  JQ798384 | JQ798366  JQ798367  JQ798368 | Tibet, China | 6 |
| *M. sinomaculata* | KM044460 | KM044493 | Sichuan, China | 2 |
| *M. sinuata* | AY328268 | AY328216 | Yuan, 2002 | 1 |
| *M. smithiana* | AY328301 | AY328247 | Yuan, 2002 | 1 |
| *M. speciosa* | KM044464  AY328286 | KM044497  AY328220 | Tibet, China  Yuan, 2002 | 2 |
| *M. superba* | AY328274 | AY328225 | Yuan, 2002 | 1 |
| *M. sp1* | KM044461  KM044462 | KM044494 KM044495 | Sichuan, China | 2 |
| *M. sp2* | KM044464 | KM044496 | Tibet 71 |  |
| *M. taylorii* | AY328275 | N/A | Yuan, 2002 | 1 |
| *M. torquata* | AY328278 | AY328222 | Yuan, 2002 | 1 |
| *M. villosa* | AY328302 | AY328245 | Yuan, 2002 | 1 |
| *M. wilsonii* | KM044465 | KF513555 | Sichuan, China | 2 |
| *M. wumungensis* | AY328265 | AY328214 | Yuan, 2002 | 1 |
| *M. zangnanensis* | KM044466 | KF513556 | Tibet, China | 3 |
| *P. aculeatum* | DQ250316  DQ250317 | DQ251167  DQ251168 | Carolan *et al.*, 2006 | 2 |
| *P. alboroseum* | AB490306AB490307  AB490308 | N/A | Yamagishi *et al.*, 2010 | 3 |
| *P. alpinum* | JX079023  DQ250261  DQ250268 | DQ251119  DQ251112 | Carolan *et al.*, 2006  Xiao, unpub. | 3 |
| *P. anomalum* | DQ250264  DQ250263 | N/A | Carolan *et al.*, 2006 | 2 |
| *P. apulum* | DQ250300 | DQ251151 | Carolan *et al.*, 2006 | 1 |
| *P. argemone* | DQ250298 | DQ251149 | Carolan *et al.*, 2006 | 1 |
| *P. armeniacum* | DQ250259  DQ250294  DQ250297  DQ250302  DQ250311  DQ250312 | DQ251145  DQ251148  DQ251153  DQ251162  DQ251163 | Carolan *et al.*, 2006 | 6 |
| *P. atlanticum* | DQ250293  DQ250303  DQ250307  DQ250315  JF774161 | DQ251144  DQ251154  DQ251158  DQ251166 | Carolan *et al.*, 2006  Valtuena *et al.*, 2011 | 5 |
| *P. bracteatum* | DQ912881DQ250286  DQ250287 | DQ251137  DQ251138 | Carolan *et al*,*.* 2006 | 3 |
| *P. californicum* | DQ250318 | DQ251169 | Carolan *et al.*, 2006 | 1 |
| *P. commutatum* | DQ250313 | DQ251164 | Carolan *et al.*, 2006 | 1 |
| *P. croceum* | DQ250257  DQ250258 | N/A | Carolan *et al.*, 2006 | 2 |
| *P. dubium* | DQ250267 DQ250319  DQ250322 | DQ251118  DQ251121  DQ251170  DQ251173 | Carolan *et al.*, 2006 | 4 |
| *P. fauriei* | AB490302 | N/A | Yamagishi *et al.*, 2010 | 1 |
| *P. glaucum* | DQ250308DQ250309  DQ250310 | DQ251159  DQ251160  DQ251161 | Carolan *et al.*, 2006 | 3 |
| *P. hybridum* | DQ250301 | DQ251152 | Carolan *et al.*, 2006 | 1 |
| *P. lateritium* | JX078983 | N/A | Xiao, unpub. | 1 |
| *P. macrostomum* | DQ250275 | DQ251126 | Carolan *et al.*, 2006 | 1 |
| *P. miyabeanum* | DQ250265DQ250276 | N/A | Carolan *et al.*, 2006 | 2 |
| *P. nudicaule* | KM044467  AB490304  AB490305  AB490313  DQ250260DQ250284 | KF513557  DQ251116  DQ251117  DQ251127  DQ251135 | Beijing, China  Carolan *et al.*, 2006  Yamagishi *et al.*, 2010 | 5 |
| *P. nudicaule var.*  *aquilegioides* | N/A | DQ251115 | Carolan *et al.*, 2006 | 1 |
| *P. orientale* | DQ912886  DQ250290  DQ250291 | DQ251140  DQ251141  DQ251142  DQ251143 | Carolan *et al.*, 2006  Lee *et al.*, 2010 | 5 |
| *P. pavoninum* | KM044468  KM044469  DQ250283 | KM044498  DQ251134 | Xinjiang, China  Carolan *et al.,* 2006 | 5 |
| *P. pilosum* | DQ250320  DQ250321 | DQ251171  DQ251172 | Carolan *et al.,* 2006 | 2 |
| *P. setiferum* | DQ250285  DQ250288  DQ250296 | DQ251120  DQ251139  DQ251147 | Carolan *et al.,* 2006 | 3 |
| *P. radicatum* | KM044470  KM044471  DQ250262 | KF513558  DQ251113 | Jilin, China 05,06  Carolan *et al.,* 2006 | 3 |
| *P. rhoeas* | DQ250273  DQ912886  FJ469600  AF098920 | AY328240  DQ251124  DQ251123 | Carolan *et al.,* 2006  Schwarzbach and Kadereit, unpub.  Lee and Yang, unpub.  Yuan, 2002 | 4 |
| *P. somniferum* | DQ250280DQ250281  DQ250282  DQ250304  DQ250305  DQ250306  DQ364699DQ912882  JN584660 | DQ251131  DQ251132DQ251155  DQ251156  DQ251157  AY328242 | Yuan, 2002  Quesada-Moraga *et al.*, 2006  Lee *et al.,* 2010  Lee and Choe, unpub. | 9 |
| *P. somniferum*  *subsp. setigerum* | JN584656  JN584657JN584658  JN584659 | JN584678 | Lee and Choe, unpub. | 4 |
| *P. sp* | AB490314 | AY328241 | Yuan, 2002  Yamagishi *et al.,* 2010 | 1 |
| *P. sp.* | JX079012 | N/A | Xiao, unpub. | 1 |
| *P. sp.* | AB490312 | N/A | Yamagishi *et al.,* 2010 | 1 |
| *P. sp.* | AB490310 | N/A | Yamagishi *et al.,* 2010 | 1 |
| *P. sp.* | AB490313 | N/A | Yamagishi *et al.,* 2010 | 1 |
| *P. sp.* | AB490311 | N/A | Yamagishi *et al.,* 2010 | 1 |
| *P. sp.* | AY328298 | N/A | Yuan, 2002 | 1 |
| *P. spicatum* | AY328296 | AY328244 | Yuan, 2002 | 1 |
| *P. triniaefolium* | N/A | AM397153 | Worberg *et al.*, 2007 | 1 |
| *Argemone albiflora* | JX078976 | N/A | Xiao, unpub. | 1 |
| *A. mexicana* | AY328303 | AY328248 | Yuan, 2002 | 1 |
| *Chelidonium majus* | JX079037 | AY328251 | Yuan, 2002; Xiao, unpub. | 1 |
| *Dendromecon harfordii* | JF892633 | JF892706 | Still and Potter, 2013 | 1 |
| *D. rigida* | JF892635 | JF892708 | Still and Potter, 2013 | 1 |
| *Dicranostigma franchetianum* | AY328305 | AY328250 | Yuan, 2002 | 1 |
| *D. lactucoides* | AY328304 | AY328249 | Yuan, 2002 | 1 |
| *Discocapnos mundtii* | HE603316 | HE603345 | Perez-Gutierrez *et al.*, 2012 | 1 |
| *Eschscholzia rhombipetala* | JF892632 | JF892705 | Lee *et al.*, 2010 | 1 |
| *E. californica_* | DQ912883 | JF892673 | Lee *et al.*, 2010  Still and Potter, 2013 | 1 |
| *Fumaria capreolata* | HE603307 | HE603328 | Perez-Gutierrez *et al.*, 2012 | 1 |
| *Glaucium flavum* | HQ993100 | N/A | Heo and Suh, unpub. | 1 |
| *Hunnemannia fumariifolia* | N/A | JF892709 | Lee *et al.*, 2010 | 1 |
| *H. hintoniorum* | JF892637  JF892638 | JF892711 | Lee *et al.*, 2010 | 2 |
| *Hylomecon hylomeconoides* | DQ912876 | N/A | Lee *et al.*, 2010 | 1 |
| *H. japonica* | N/A | FJ626563 | Wang, 2009 | 1 |
| *Macleaya*  *microcarpa* | N/A | FJ626564 | Wang, 2009 | 1 |
| *M. cordata* | AY328307 | N/A | Yuan, 2002 | 1 |
| *Roemeria refracta* | KM044472  DQ250299 | KM044499 | Xingjiang-35  Carolan *et al.,* 2006 | 2 |
| *Stylomecon heterophylla* | DQ250295 | DQ251146 | Carolan *et al.,* 2006 | 1 |
| *Stylophorum diphyllum* | *AY328309* | *AY328252* | *Yuan, 2002* | 1 |

N: indiviadual number

**LITERATURE CITED**

Carolan JC, Hook IL, Chase MW, Kadereit JW, Hodkinson TR. 2006. Phylogenetics of *Papaver* and related genera based on DNA sequences from ITS nuclear ribosomal DNA and plastid *trnL* intron and *trnL–F* intergenic spacers. Annals of Botany 98: 141–155.

Heo K, Suh Y. Molecular phylogeny of Chelidoniodeae based on ITS sequences. Unpublished.

Lee EJ, Choe SG. Species identification of *Papaver setigerum* naturalized in Korea by genetic and chemical components analysis. Unpublished.

Lee EJ, Hwang IK, Kim NY, Lee KL, Han MS, Lee YH, Kim MY, Yang MS. 2010. An assessment of the utility of universal and specific genetic markers for opium poppy identification. [Journal of Forensic Sciences](https://www.google.com.hk/url?sa=t&rct=j&q=&esrc=s&source=web&cd=1&cad=rja&ved=0CCYQFjAA&url=http%3A%2F%2Fonlinelibrary.wiley.com%2Fjournal%2F10.1111%2F(ISSN)1556-4029&ei=1eQWU9SDD6WTiQfS34DwAg&usg=AFQjCNF1TY_YhC7KDJqKFZYXChmhnv04tQ&bvm=bv.62286460,d.aGc) 55: 1202–1208.

Lee EJ, Yang MS. The discrimination study of poppy families. Unpublished.

Perez-Gutierrez MA, Romero-Garcia AT, Salinas MJ, Blanca G, Fernandez MC, Suarez-Santiago VN. 2012. Phylogeny of the tribe Fumarieae (Papaveraceae s.l.) based on chloroplast and nuclear DNA sequences: Evolutionary and biogeographic implications. American Journal of Botany 99: 517–528.

Quesada-Moraga E, Landa BB, Munoz-Ledesma J, Jimenez-Diaz RM, Santiago-Alvarez C. 2006. Endophytic colonisation of opium poppy, *Papaver somniferum*, by an entomopathogenic Beauveria bassiana strain. Mycopathologia 161: 323–329.

Schwarzbach AE, Kadereit JW. Phylogeny of prickly poppies (*Argemone* L.: Papaveraceae) and the evolution of morphological and alkaloid characters based on ITS nrDNA sequence variation. Unpublished.

Still SM, Potter D. 2013. California poppy conundrums: insight into the relationships within the tribe Eschscholtzieae (Papaveraceae). Systematic Botany 38: 104–117.

Valtuena FJ, Preston CD, Kadereit JW. 2011. Evolutionary significance of the invasion of introduced populations into the native range of *Meconopsis cambrica*. Molecular Ecology 20: 4318–4331.

Wang W, Lu AM, Ren Y, Endress ME, Chen ZD. 2009. Phylogeny and classification of Ranunculales: Evidence from four molecular loci and morphological data. Perspectives in Plant Ecology, Evolution and Systematics 11: 81–110.

Worberg A, Quandt D, Barniske A-M, Löhne C, Hilu KW, Borsch T. 2007. Phylogeny of basal eudicots: Insights from non-coding and rapidly evolving DNA. Organisms Diversity & Evolution 7: 55–77.

Xiao W. Phylogenetics and Biogeography of *Meconopsis* (Papaveraceae). Unpublished

Yamagishi M, Yoshida E, Aikoh T, Kondo T, Takahashi H. 2010. A cultivated poppy *Papaver* sp.) invades wild habitats of *Papaver fauriei* in the mountain area of Rishiri Island, Japan. Landscape and Ecological Engineering 6: 155–159.

Yuan CC. 2002. The phylogeny, systematics and biogeography of *Meconopsis* Vig. (Papaveraceae) and *Craigia* W. W. Sm. & W. E. Evans (Tiliaceae). PH. D. Dissertation. Schools of Life Science, Sun Yat-Sen University. Guangzhou, China.
