# Supplementary material for: Molecular Phylogeny of Asian Meconopsis Based on Nuclear Ribosomal and Chloroplast DNA Sequence Data
Source: PLoS One. 2014 Aug 12;9(8):e104823. doi: 10.1371/journal.pone.0104823 (PMC4130606; doi:10.1371/journal.pone.0104823)
Supplement: Table S2 — Classifications of Meconopsis by Taylor [1] and Wu & Chuang [2] . (DOC) [file pone.0104823.s002.doc]

**Table S2. Classifications of *Meconopsis* by Taylor [1] and Wu & Chuang [2]**

| ***Taylor 1934*** | | | | Wu & Chuang 1980 | | |
| --- | --- | --- | --- | --- | --- | --- |
| ***Subgen.*** | ***Sect.*** | ***Subsect.*** | ***Series and species*** | ***Subgen.*** | ***Sect.*** | Series and species |
| **Eumeconopsis** | Cambricae |  | M. cambrica | **Meconopsis** | **Cambricae** | Ser. *Cambricae*  ***M. cambrica***  ***M. dhwojii***  ***M. gracilipes***  ***M. longipetiolata***  ***M. napaulensis***  ***M. paniculata***  ***M. regia***  ***M. taylorii***  Ser. *Chelidonifoliae*  ***M. chelidonifolia***  ***M. oliveriana*** |
|  | **Eucathcartia** |  | ***Ser.* Chelidonifoliae**  M. chelidonifolia  M. oliveriana |  |  |  |
|  |  |  | ***Ser.* Villosae**  M. smithana  M. villosa |  |  |  |
|  | **Polychaetia** | **Eupolyc**-**haetia** | ***Ser.* Superbae**  M. regia  M. superba  ***Ser.* Robustae**  M. dhwojii  M. gracilipes  M. longipetiolata  M. napaulensis  M. paniculata  M. robusta  M. violacea |  | **Racemosae** | Ser. *Grandes*  ***M. betonicifolia***  ***M. grandis***  ***M. integrifolia***  ***M. smithana***  ***M. villosa***  Ser. *Racemosae*  ***M. aculeata***  ***M. florindae***  ***M. georgei***  ***M. latifolia***  ***M. lyrata***  ***M. primulina***  ***M. racemosa***  ***M. robusta***  ***M. sinuata***  ***M. speciosa***  ***M. superba***  ***M. violacea*** |
|  |  | **Cummin-sia** | ***Ser.* Simplicifoliae**  M. punicea  M. quintuplinervia  M. simplicifolia  ***Ser.* Grandes**  M. betonicifolia  M. grandis  M. integrifolia |  | **Simplicifoliae** | Ser. *Simplicifoliae*  ***M. punicea***  ***M. quintuplinervia***  ***M. simplicifolia*** |
|  |  |  |  |  | **Forrestii** | Ser. *Forrestii*  ***M. forrestii***  ***M. lancifolia*** |
|  |  |  | ***Ser.* Primulinae**  M. florindae  M. lyrata  M. primulina  ***Ser.* Delavayanae**  M. delavayi  ***Ser.* Aculeatae**  M. aculeata  M. forrestii  M. georgei  M. henrici  M. horridula  M. impedita  M. lancifolia  M. latifolia  M. neglecta  M. pseudovenusta  M. sinuata  M. speciosa  M. venusta |  |  | Ser. *Henricanae*  ***M. barbiseta***  ***M. concinna***  ***M. henrici***  ***M. neglecta***  ***M. wumungensis*** |
|  |  |  |  |  |  | Ser. *Delavayanae*  ***M. argemonantha***  ***M. bella***  ***M. delavayi***  ***M. horridula***  ***M. impedita***  ***M. pseudohorridula***  ***M. pseudovenusta***  ***M. zangnanensis*** |
|  | *undetermined* |  | M. argemonantha |  |  |  |
|  | *undetermined* |  | *S****er.* Bellae**  M. bella |  |  |  |
| **Discogyne** |  |  | M. discigera  M. torquata | **Discogyne** | **Discogyne** | Ser. *Discogyne*  ***M. discigera***  ***M. pinnatifolia***  ***M. torquata*** |
